# Environmental health risk assessment of sulfur dioxide (SO_2_) at workers around in combined cycle power plant (CCPP)

**DOI:** 10.1016/j.heliyon.2022.e09388

**Published:** 2022-05-06

**Authors:** Shofi Nurhisanah, Hamzah Hasyim

**Affiliations:** Department of Environmental Health, Faculty of Public Health, Universitas Sriwijaya, Indonesia

**Keywords:** Environmental health risk assessment (EHRA), Sulfur dioxide, Risk quotient (RQ), Worker

## Abstract

State electricity company is an Indonesian government-owned corporation with a monopoly on Indonesia's electricity distribution. Sulfur dioxide (SO_2_) pollution is produced by burning fossil fuels with coal and oil-fired power plants. At the combined cycle power plant (CCPP), the state electricity company has the largest role in SO_2_ production. In addition, SO_2_ can cause respiratory tract dysfunction, decreased lung function, eye irritation, throat irritation, and coughing at certain concentrations. This study aims to assess the magnitude of SO_2_ exposure to workers health at CCPP Indralaya unit, Indonesia. The research is a quantitative study using the environmental health risk assessment (EHRA) method. Purposive sampling was used to obtain 32 respondents. The results revealed that the average SO_2_ concentration was 0.085 mg/m^3^. The non-carcinogenic intake was 0.0025 mg/kg/day for real-time exposure and 0.0069 mg/kg/day for lifetime exposure. The Risk Quotient (RQ) for real-time exposure obtained is 0.0959, and RQ for lifetime exposure is 0.2668, indicating an RQ = 1. The study concluded that the CCPP Indralaya unit is not at-risk cause non-carcinogenic due to SO_2_ exposure. Regardless, precautions must ensure that workers' exposure to SO_2_ or other emissions gases produced by CCPP activities does not endanger their health.

## 1 Introduction

According to World Health Organisation (WHO), approximately 7 million people died due to airborne pollutants, with an estimated 200 thousand deaths due to outdoor pollution in urban areas, with around 93 per cent of cases occurring in developing countries (WHO, 2014). Short-term Sulfur dioxide (SO_2_) exposure has been linked to respiratory morbidity in adults and children, especially asthmatic and elderly populations. Furthermore, there are intermittent spikes in SO_2_ concentrations, which may harm health (Anastasopolos et al., 2021). Based on data from 272 Chinese cities, Wang and Liu measured the health effects of SO_2_ exposure. They estimated that the SO_2_ concentration occurred at 10 g/m^3^ and that a two-day increase in the mean SO_2_ concentration resulted in a 0.59 per cent increase in mortality (Wang et al., 2018). It is considered a significant air pollutant, especially in developing countries, causing health problems (Serbula et al., 2021).

Sulfur dioxide is harmful to human health, particularly respiratory and lung functions. People who work seven days a week with no days off are at a high risk of SO_2_ poisoning when levels are high (Wijiarti et al., 2016). Concerns about the health risks posed by SO_2_ pollution prompted a risk assessment for a heavily polluted industrial region in South Africa (Matooane and Diab, 2003). According to the Material Safety Data Sheet (MSDS), at 20 ppm, SO_2_ gas exposure can cause eye irritation, nose, throat, sinuses, pulmonary oedema, and even death. Another negative impact of this pollutant on humans is respiratory tract irritation and decreased lung function, which results in coughing, shortness of breath, and asthma (Muziansyah et al., 2015). Emissions can spread in response to meteorological conditions, such as wind direction and fluctuations in turbulence and atmospheric stability, which are highly dynamic on a temporal and spatial scale and can quickly harm health (Turyanti et al., 2016). Residents who live within a 300-meter radius of industrial areas have a 1.37-fold risk of reduced lung function capacity and a 1.62 - fold risk of reduced lung function (Daud, 2013).

Sulfur dioxide concentration continues to rise with the increased use of fossil fuels. According to Solichin, SO_2_ from natural gas-fired power plants accounts for 38.8 per cent of the total, exceeded only by coal (Solichin, 2016). The Combined Cycle Power Plant (CCPP) located at the keramasan sector, Indralaya unit, Ogan Ilir District, Indonesia, is a gas and gas-steam power plant operation. According to the air quality analysis and work environment monitoring results in the first quarter of 2020, the average level of SO_2_ pollution was 53.33 g/Nm^3^/1 h during monitoring at 9 points around the CCPP. This level failed the SO_2_ quality standard. The measurement of SO_2_ concentration, on the other hand, is rising annually. It is a health risk because, in this case, several CCPP workers at Indralaya complained about sore eyes and coughing when working near sources during a preliminary survey. As a result, it is necessary to research the Environmental Health Risk Assessment (EHRA) of SO_2_ exposure to the state electricity company's workers. Environmental Health Risk Assessment is increasingly used in public health decision-making, environmental regulation, and research planning (WHO, 2000). According to the National Academy of Sciences (NRC) report, any risk assessment must include four steps, namely: *hazard identification, dose-response analysis, exposure assessment, and risk characterisation* (Louvar and Louvar, 1998; WHO, 2000). Besides, Environmental Health Australia (EHA) formalised EHRA, adding it to five stages, where the first stage is issue identification (Enhealth, 2021).

## 2 Materials and methods

This research is a quantitative study with a descriptive research design that employs the EHRA method to assess human health risks from environmental hazards.

First, the mean, minimum, and maximum values for SO_2_ concentration data, age, activity pattern data, and anthropometric data are determined using frequency distribution analysis. Then, to calculate the amount of intake received by an individual, a health risk analysis calculates the SO_2_ exposure intake of respondents. Intake is calculated using anthropometric data, frequency of exposure, and duration of exposure for each respondent, and the value of intake is calculated using the average value of all variables. Researchers conducted SO_2_ measurements in the morning and afternoon with Palembang Environmental Health and Disease Control Engineering Center experts. These measurements are taken at four locations throughout the work area using a vacuum pump and an impinger tube. In the CCPP Indralaya study area, direct measurement is used to collect SO_2_ concentrations in the workplace. Measurements of SO_2_ here were conducted in four different locations, namely, Medco's Matering Gas area, ST 1.0 Control room area, Cooling Tower area, and Water Treatment Plant area. The power generation capacity of the CCPP plant consists of one unit of Gas Turbine Power Plant and one unit of Steam-electric Power Plant. The fuel is natural gas lubricants, Shell Turbo Oil T-46.

The population for this study was all CCPP Indralaya unit employees who worked in SO_2_ measurement, and the sample size was 32 respondents. The purposive sampling technique was used, and the inclusion criteria were workers who had been around the work area for 8 h or more, had worked in the company for one year or more, were aged 20 years and over, and had a minimum bodyweight of 50 kgs.

The formula employed in this study was (Louvar and Louvar, 1998; ATSDR, 2005).

The Agency for Toxic Substances and Disease Registry (ATSDR), headquartered in Atlanta, Georgia, is a federal public health agency.

Intake formula:

(1) $$I_{nk}=\frac{C\;X\;R\;X\;t_{E}\;X\;f_{E}\;X\;D_{t}}{W_{b}\;X\;t_{avg}}$$

RQ formula:

(2) $$\;\text{RQ}=\frac{I_{nk}}{RfD\;orRfC}$$

Information:

I_nk_ = *Intakes* (mg/kg/day)
C = Concentration (mg/m^3^)
R = Inhalation rate (0.83 m^3^/hour)
t_E_ = Time of exposure (hours/day)
*f*_E_ = Frequency of exposure (days/year)
W_b_ = Weight of body (kg)
D_t_ = Duration time, real time or 30 years projection
t_avg_ = Time average period (30 years × 365 days/year for non-carcinogenic substances)
RfC = Reference concentration (mg/kg/day)
RQ = *Risk Quotient*

Anthropometric characteristics are the workers' bodyweight, measured directly during the interview using a weight scale. In addition, the pattern of worker activity, which includes exposure time (t_E_), exposure frequency (*f*_E_), and exposure duration (*Dt*), was obtained through direct interviews with workers using questionnaires.

The study received Ethical Approval (No:361/UN9.1.10/KKE/2020) from the Health Research Ethics Committee Faculty of Public Health, Sriwijaya University. Participation was voluntary, and there was no financial incentive.

## 3 Results

The data were analysed using univariate analysis, which aims to explain the characteristics of each variable, such as age, the highest level of education, bodyweight, exposure time, exposure frequency, and duration of exposure. In addition, EHRA was used to determine the magnitude of the risk generated by each worker. The distribution of characteristics of respondents is shown in Table 1.

**Table 1:** Distribution frequency of worker characteristics.

Variable Characteristics of Respondents		Frequency	Percentage
Gender	Male	32	100
	Female	0	0
	**Total**	**32**	**100**
Age	<40 Years	18	56.4
	≥40 Years	14	43.6
	**Total**	**32**	**100**
Level of Education	Primary School	1	3.1
	Junior High School	5	15.6
	Senior High School	12	37.5
	Diplom/Bachelor	14	43.8
	**Total**	**32**	**100**

Table 1 shows that 32 of the respondents who work as CCPP employees are male. More than half the respondents were aged less than 40 years (N = 18, 56.4%), and the remaining were aged 40 or more years (N = 14, 43.6 per cent). The highest education level was at Diploma/Bachelor level (N = 14, 43.8 per cent). The education level is included as this variable may also be related to risk. For example, low education levels contribute to workers' ignorance of the dangers of SO_2_ inhalation. It is hypothesised that the risk of developing respiratory complaints will be increased in this group.

Table 2 shows the highest SO_2_ concentration measurement point 4 results in the Water Treatment Plant (WTP) area, with a morning measurement time of 0.1172 mg/m^3^. Meanwhile, the lowest point 2 is in the control room area, with a morning measurement of 0.0518 mg/m^3^. Sulfur dioxide concentration is still a safe limit according to the threshold limit value (TLV) according to the regulation of the Ministry of Manpower and Transmigration in Indonesia (Permenakertrans) No. Per.13/MEN//X/2011 concerning the workplace's threshold value of physical and chemical factors. The maximum allowable is 2 mg/m^3^.

**Table 2:** A frequency distribution of sulfur dioxide (SO_2_) concentration.

No	Sampling Point	SO_2_ Concentration			Average SO_2_ Concentration (mg/m^3^)
		Time	Temperature and Humidity	SO_2_ Concentration (mg/m^3^)	
1.	Medco's Matering Gas	In the Morning (09.15 am)	T = 28.4 °C H = 38.2%	0.0927	0.0956
		In the Afternoon (01.07 pm)	T = 32.5 °C H = 64%	0.0986	
2.	ST 1.0 Control room	In the Morning (09.28 am)	T = 28.5 °C H = 65.9%	0.0518	0.0524
		In the Afternoon (01.11 pm)	T = 32.5 °C H = 64%	0.0530	
3.	Cooling Tower	In the Morning (10.12 am)	T = 33 °C H = 77%	0.0804	0.0862
		In the Afternoon (02.00 pm)	T = 34.9 °C H = 69% ^o^C	0.0919	
4.	Water Treatment Plant (WTP)	In the Morning (10.19 am)	T = 33.1 H = 77%	0.1172	0.1094
		In the Afternoon (02.00 pm)	T = 34.9 °C H = 69%	0.1015	

Table 3 shows the Kolmogorov Smirnov test for SO_2_ concentration, weight, daily exposure, frequency of exposure, exposure duration and intake of SO_2_ in real-time and over a lifetime. Furthermore, the table shows that the average SO_2_ concentration is 0.085 mg/m^3^, with a median value of 0.090 mg/m^3^. Additionally, the ambient air contains a minimum SO_2_ concentration of 0.0524 mg/m^3^ and a maximum SO_2_ concentration of 0.1094 mg/m^3^. In addition, the bodyweight distribution of workers CCCP is 63.44 kg, with a median value of 65 kg.

**Table 3:** A frequency distribution analysis.

Variable	Mean	Median	SD	Min	Max	p-value
**SO**_**2**_**Concentration**						
*Sulfur Dioxide Concentration*	0.085	0.090	0.0213	0.0524	0.1094	0.031
**Anthropometric Characteristics**						
*Weight*	63.44	65.00	6.420	50	73	0.199
**Activities Pattern**						
Exposure Time	8.44	8.00	0.840	8	10	0.001
*Frequency of Exposure*	265.22	242.00	41.963	242	343	0.001
*Exposure Duration*	11.88	11.00	4.824	2	25	0.167
**Intake Calculation**						
*Intake Realtime* (mg/kg/day)	0.002506	0.002450	0.0012560	0.0003	0.0058	0.991
*Intake Lifetime* (mg/kg/day)	0.006938	0.006750	0.0024210	0.0032	0.0114	0.907

The average exposure time for workers is 8.44 h/day, with most workers having a t_E_ of less than or equal to 8 h per day for as many as 25 workers. The annual frequency of exposure is 265.22 days/year, with most workers having an *f*_E_ of less than or equal 242 days for as many as 22 workers. The exposure duration is 11.88 years, with most workers having a t_E_ of less than or equal to 242 days for as many as 22 workers. Then in intake calculation, the average intake or real-time exposure intake for CCPP Indralaya workers is 0.0025 mg/kg/day; the average lifetime exposure intake is 0.0069 mg/kg/day. As many as 17 workers have a real-time intake value of 0.0025 mg/kg/day. In addition, 18 workers have an intake lifetime value of 0.0069 mg/kg/day.

**Figure 1:**
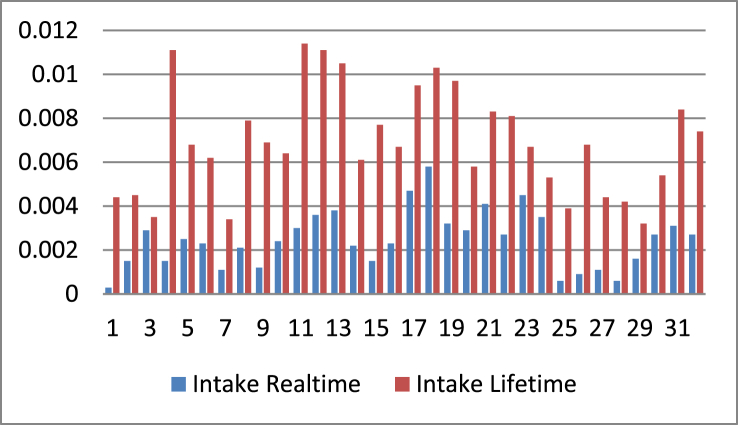
Distribution analysis of intake of SO_2_ for realtime and lifetime exposure.

According to Figure 1, the essential real-time intake value is found in 18 respondents with an exposure duration of 17 years with a bodyweight of 65 kg, which is 0.0058 mg/kg/day. The essential lifetime intake value is found in 11 respondents aged 34 years and bodyweight of 65 kg Figure 1 depicts the results of the calculation of the real-time and lifetime intake values for 32 respondents:

Furthermore, the RQ Distribution Analysis is shown in Table 4.

**Table 4:** Distribution analysis of RQ.

Variable	Mean	Median	SD	Min	Max	p-value
RQ *Realtime* (mg/kg/day)	0.0959	0.0942	0.0486	0.0115	0.2231	0.878
RQ *Lifetime* (mg/kg/day)	0.2668	0.2596	0.0931	0.1231	0.4385	0.907

**Figure 2:**
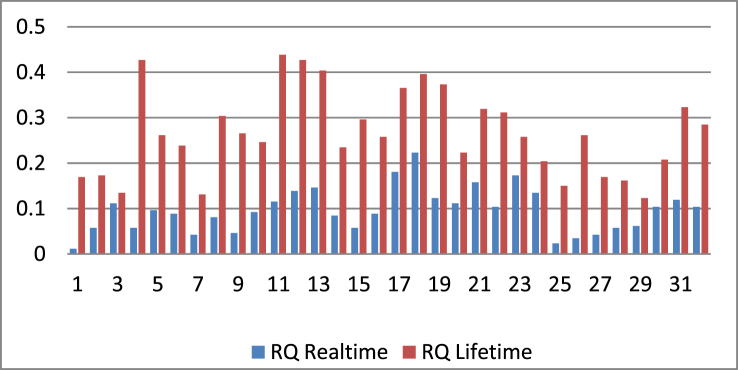
RQ of SO_2_ for realtime and lifetime exposure.

According to Table 4, the average Risk Quotient (RQ) for real-time exposure to CCPP workers is 0.095 mg/kg/day. The RQ for a moderate lifetime exposure is 0.2668 mg/kg/day, according to the results of the overall calculation for respondents for real-time and lifetime exposure. There are no respondents with greater than or equal to one (RQ > 1), so the risk to workers at this time can still be considered no risk. The results of the RQ calculation on the respondents are shown in Figure 2 below:

According to the graph of risk characteristics of SO_2_ for workers for real-time exposure (Figure 2), the highest risk level occurred in 18 respondents with an exposure duration of 17 years, which was 0.2231 mg/kg/day. Meanwhile, the highest lifetime risk level was 0.4385 mg/kg/day, which occurred in 11 respondents with an exposure duration of 8 years. In addition, the expectation of risk is shown in Table 5.

**Table 5:** Risks are expected to be high in the fifth, tenth, fifteenth, twentieth, twenty-fifth, twenty-fifth, and thirty-first years.

	D_t_ -5	D_t_ -10	D_t_ -15	D_t_ -20	D_t_ -25	D_t_ -30
**RQ**	0.0427	0.0877	0.1330	0.1778	0.2221	0.2668

Table 5 shows the estimated non-carcinogenic risk (RQ) of exposure to SO_2_ in ambient air for CCPP over the next 5^th^, 10^th^, 15^th^, 20^th^, 25^th^, and 30^th^ years. It aims to determine the significant increase in risk per duration of exposure ranging from 5 to 30 years. Calculated risk is the risk in five years. The intake calculation is first performed by substituting the numbers 5, 10, 15, 20, 25, and 30 years for the duration of exposure to calculate the RQ every five years. Then, the RQ of each intake result is calculated, and the RQ value for each of the following five years is recorded in the table.

A few workers indicated that they reported some signs and symptoms of illness, specifically non-communicable diseases such as gout, ulcers, and asthma. However, this study did not ask about the types of comorbidities. CCPP collaborates with external health care providers to conduct employee health checks, and CCPP's health insurance covers all employees. Additionally, CCPP central conduct routine health checks on all employees every two months or six months, bringing doctors or other health care professionals to the company. Historically, a control hierarchy has been used to determine the most feasible and effective control solutions. Among them were administrative controls and personal protective equipment.

## 4 Discussion

Based on the research findings on the analysis of SO_2_ concentrations on workers, measurements were taken at four different locations noted above. Point 4 (WTP area) recorded the highest value as 0.109 mg/m^3^. The lowest value was 0.052 mg/m^3^ at point 2 of the control room area. However, the SO_2_ concentration results did not exceed the threshold set by Permenakertrans No. 13 of 2011. Wahyuddin stated that exposure to SO_2_ that occurred in the traffic police of Surakarta could cause lung problems with SO_2_ concentrations that were small or below the threshold value (Wahyuddin et al., 2016). If exposed regularly, this will cause respiratory complaints ranging from coughing up phlegm, shortness of breath, and dry cough, to a sore throat (Sandra, 2013; Wahyuddin et al., 2016).

Similarly, Solichin conducted a study in the power plant and boiler area of PT. Pusri Palembang with an SO_2_ concentration of 0.246 mg/m^3^ (Solichin, 2016). The significant difference in concentration between this study and other studies is differences in the source of the SO_2_ pollutant itself. The concentration difference is substantial because different studies are sourced from mobile sources such as line sources (roads) and area sources (bus terminals). In contrast, the SO_2_ pollutant source in this study is from a stationary source, specifically the CCPP. This source is one of the most important contributors to global SO_2_ emissions caused by human activities, namely coal, gas, and oil as the primary fuel. Aside from the natural and artificial sources, it is no surprise that SO_2_ is also found in food and beverages consumed (Peng et al., 2014). Investigated the residual sulphur dioxide content of 2116 samples from nine foods and discovered that vegetables and fruits had relatively high levels.

### 4.1 Participant characteristics

According to the research findings conducted through interviews and questionnaires, the characteristics of respondents in CCPP are known to all workers who meet the inclusion criteria, with 100 per cent being male. The age of respondents was divided into two categories: under 40 years and 40 or more years. More than half were under 40 years old. The calculation of intake is proportional to the duration of exposure and the respondent's age. The intake value is affected by the respondent's age; the older the respondent, the longer the respondent's exposure, and the higher the intake value generated. Age can affect the body's resistance to toxic substances or chemicals, whereby ageing reduces physiological functions increases the risk of health problems (Meo et al., 2013; Mukono, 2009; Zaenurrohmah and Rachmayanti, 2017).

According to the study's findings, the workers' bodyweight ranged from 50 kg to 73 kg, with an average bodyweight of 63.44 kg. The formula calculation's weight value is the denumerator, so the result is proportional to the intake. Respondents with a significant bodyweight face a low risk, and vice versa; the lower the risk, the higher the value of the intake calculation. The respiratory system's work is heavier in people with significant bodyweight, and lung capacity is relatively smaller than in people with a lightweight. The greater the volume of a person's lungs into which SO_2_-containing air enters, the greater the possibility of jeopardising the person's health. Furthermore, everyone's weight has a different value due to various factors such as nutrition, consumption patterns, culture, hormones, and the environment.

Air weighing 55 kg, according to Nukman, can be considered a usual adult Indonesian standard as long as no more comprehensive study of anthropometric characteristics is conducted (Nukman et al., 2019). It is assumed that respondents do not consider their lifestyle and intake patterns while at work; on the exposure time variable, the researchers discovered that not all respondents set aside some time to rest. Furthermore, respondents with the healthiest bodyweight have a large lung volume capacity, allowing more air to enter the body and increasing the likelihood of breathing air containing SO_2_.

### 4.2 Intake rate

Unlike bodyweight data, interviews or direct measurements cannot determine intake rate (R). Bodyweight is a determinant of the oxygen demand of the air that must be inhaled. Inhalation rates and bodyweights predict high-end exposures for individuals (International Programme On Chemical, 2008). So, that the rate of inhalation is a function of bodyweight in addition to age, gender, and activity patterns, the equation y = 5.3 Ln(x)-6.9 is used to calculate the relationship between bodyweight and intake rate, where y = R unit m^3^/day and x = Wb or bodyweight. If we apply this equation to the respondent's weight (WHO, 2014), which is 51 kg, the inhalation rate is R = 13.65 m^3^/day or 0.57 m^3^/hour. This figure is 68 per cent of the US-determined EPA's value of the inhalation rate (R), which is 0.83 m^3^/hour, making this equation more appropriate for toddlers and children. Based on this, and the fact that the average bodyweight of workers is 63.44 kg, the intake rate (R) in this study continues to use the US Environmental Protection Agency (US-EPA) determination value of 0.83 m^3^/hour.

### 4.3 Exposure time

EPA and Permenakertrans No. 13 of 2011 recommend only 8 h of work per day. If the SO_2_ concentration remains below the threshold value, the exposure time in this study is still considered no risk. The exposure time in research had a median value of 24 h/day of exposure (Ma'rufi, 2018). According to the study, share the same research area and respondents, namely the source of stationary air pollutants and the factors of respondents, namely workers or adults (Novirsa and Achmadi, 2012). The longer time that is worked, the more gas is inhaled into the worker's body, and if exposed for an extended period, the respondent is more likely to be unsafe. Suppose the respondent is a permanent employee who works according to predetermined working hours. In that case, the researcher assumes a maximum exposure time in hours/day for workers in industrial areas is 8 h/day. It claims that the longer a respondent is exposed, the more likely it is to be exposed to an unsafe risk (Latifa et al., 2019). The greater the acceptable health risk, the longer a person is exposed to ammonia. It also holds for all other air pollutants, such as SO_2_.

### 4.4 Exposure frequency

According to the research findings, the frequency of exposure is an average of 265 days, ranging from 242 to 343 days. Three workers (9.4%) had an exposure frequency of 254 days per year, while seven workers (21.9%) had 343 days per year. This study's average value of exposure frequency exceeded the EPA's default value for industry exposure frequency of 250 days per year. Most employees are uncertain about their leave schedules. They may apply for leave outside of the national leave schedule and national holidays, so the frequency value of exposure to employees can change. Hoppin and Jaramillo discovered that the frequency of exposure is an essential factor in risk assessment because these variables are used to calculate the cumulative dose over time (Hoppin et al., 2011). As a result, the respondent's exposure to these substances increases with working more frequently, increasing the cumulative dose received throughout the working life. According to Harjanti and Darundiati's research, the more often a person is exposed to hazardous substances in the ambient air, the greater the health risks such as respiratory disorders (Harjanti et al., 2016).

### 4.5 Exposure duration

According to the calculation results, the real-time exposure duration ranges from 2 to 25 years, with an average Dt of 11.88 years, indicating that the average respondent has been exposed to SO_2_ from the time they started working until the study. This study is consistent with Ma'rufi findings, which had a 2-year exposure period (Ma'rufi, 2018). The duration of exposure to SO_2_ influences the health risks (Gwimbi, 2017). Because the longer a person is exposed to irritant substances, the more SO_2_ substances accumulate via the inhalation pathway and the greater the effect on the body. It is also stated that exposed workers' health status can influence health; The intensity and duration of exposure can increase health risks (Deviandhoko et al., 2013). According to this study, a respondent has a duration of exposure with a real-time RQ value of 0.115 mg/kg/day. The previous one is 25 years old. The respondent has been exposed to SO_2_ for the past 25 years. Respondents' health risks are increased as a result. According to this study, the respondents did not exceed the recommended risk level of SO_2_ exposure in the air. However, due to the various types of exposure sources, the distance between the research location and the source of exposure, and exposure concentrations that can produce varying amounts of risk, this cannot be truly proven until the risk calculation results are obtained.

### 4.6 Intake analysis

This study calculates the intake for real-time exposure (actual) and lifetime (lifelong) exposure. The value of SO_2_ intake for workers at CCPP in real-time exposure is 0.0025 mg/kg/day. At the same time, the value of SO_2_ intake for workers at CCPP over a lifetime is 0.0069 mg/kg/day. Intake is calculated using anthropometric data, frequency of exposure, and duration of exposure for each respondent, and the value of intake is calculated using the average value of all variables. As a result, the higher the value of C, t_E_*,* f_E_, and D_t_, the higher the person's intake (I). Chemical concentration, intake rate, exposure time, frequency of exposure, and duration of exposure all impact the intake value. The greater the value, the more risk agents that enter the body. Essentially, the higher the intake value of SO_2_ from exposure, the greater the respondent's risk of SO_2_. In contrast, the value of intake is also inversely proportional to bodyweight. If a person's weight is higher, the intake will be lower, and vice versa; the lower a person's weight, the higher the intake value.

### 4.7 Risk characteristics

The average risk calculation results show that a value of 0.0959 mg/kg/day is obtained in real-time exposure. The lifetime exposure risk is 0.2668, indicating that the level of SO_2_ exposure in ambient air for CCPP is classified as no or low risk. Because the RQ value is 1, the SO_2_ exposure released by CCPP industrial activity does not risk causing health effects to workers in the work area. However, this does not mean that the workers at CCPP are free of other health issues. It is consistent with Fatonah's findings that the longer the forecast time or duration of exposure (D_t_), the more respondents have an RQ > 1 (Fatonah, 2010). According to this study, respondents with the highest RQ of 0.2231 mg/kg/day have the highest intake value of 0.0058 mg/kg/day. In this study, the risk of SO_2_ exposure to workers was calculated for the next 5–30 years. The RQ generated over the next 5–30 years will increase annually, implying that the longer a worker is in an area exposed to SO_2_ emissions or has a work contract with the company, the greater the risk of SO_2_ exposure to workers.

### 4.8 Risk management

Risk management interventions include setting regulatory limits, advising on usage patterns, and controlling production at the source (WHO, 2009).

#### 4.8.1 Risk estimation

If there is a health risk for workers in a work environment, the EHRA method can formulate an effort to prevent and avoid health problems. This study does not require the risk management stage because the risk assessment is declared no or low risk at the interpretation stage. However, risk agent concentration (C) can be reduced to control the value of risk management intake. At the same time, the duration of exposure (t_E_) and exposure frequency (*f*_E_) remain the same as during the interview and for the next 30 years. Reducing contact time can be accomplished in two ways: decreasing daily exposure time (t_E_) or decreasing the frequency of exposure per year (*f*_E_) (Rahman et al., 2014). However, this is not feasible because the population in this study is workers whose work schedule and contract have been predetermined from the beginning.

#### 4.8.2 Risk management strategy

As an electricity company, the CCPP must manage critical environmental aspects in all its operations; thus, the environmental performance has been identified as a performance indicator for CCPP units throughout Indonesia to achieve a healthy environment for the company employees and the surrounding community. Specifically, this is to reduce airborne emissions that can harm health. To mitigate environmental problems caused by company activities, CCPP has implemented several environmental programmes, including waste management using the 3R's (Reuse, Reduce, Recycle), air and water pollution control.

## 5 Conclusion

The non-carcinogenic risk was calculated to be 0.0959 mg/kg/day for real-time exposure. Furthermore, the lifetime risk was 0.2668 mg/kg/day. The level of risk of SO_2_ exposure in ambient air in CCPP can be classified as safe or not at risk of causing health effects due to SO_2_ exposure for workers in the work area.

## 6 Recommendations

Despite this, efforts must be made to ensure that workers' exposure to SO_2_ or other emission gases produced by CCPP activities does not endanger their health. Workers, particularly those who serve as local operators, must be required to wear PPEappropriate to the potential hazards in the workplace, such as gloves and masks, as well as at WTP.

## Declarations

### Author contribution statement

Shofi Nurhisanah: Conceived and designed the experiments; Performed the experiments; Analyzed and interpreted the data; Contributed reagents, materials, analysis tools or data; Wrote the paper.

Hamzah Hasyim: Analyzed and interpreted the data; Wrote the paper.

### Funding statement

This research did not receive any specific grant from funding agencies in the public, commercial, or not-for-profit sectors.

### Data availability statement

The data that has been used is confidential.

### Declaration of interests statement

The authors declare no conflict of interest.

### Additional information

No additional information is available for this paper.
